# Specialty cardiac nurses’ work satisfaction is influenced by the type of coronary care unit: A mixed methods study

**DOI:** 10.1186/s12912-019-0367-6

**Published:** 2019-09-05

**Authors:** Judy Currey, Stephanie K. Sprogis, Liliana Orellana, Anusha Chander, Sharon Meagher, Rhoda Kennedy, Andrea Driscoll

**Affiliations:** 1 School of Nursing and Midwifery, Deakin University, 1 Gheringhap Street, Geelong, VIC 3220 Australia; 2 Centre for Quality and Patient Safety Research, School of Nursing and Midwifery, Deakin University, 1 Gheringhap Street, Geelong, VIC 3220 Australia; 3 Deakin Learning Futures, Office of the Deputy Vice Chancellor (Education), Deakin University, 1 Gheringhap Street, Geelong, VIC 3220 Australia; 4 Centre for Quality and Patient Safety Research – Eastern Health Partnership, 2/5 Arnold Street, Box Hill, VIC 3128 Australia; 5 Biostatistics Unit, Faculty of Health, Deakin University, 1 Gheringhap Street, Geelong, VIC 3220 Australia

**Keywords:** Coronary care units, Cardiovascular nursing, Nursing staff, Job satisfaction, Physician-nurse relations, Leadership

## Abstract

**Background:**

Many dedicated Coronary Care Units (CCUs) in Victoria, Australia, have been decommissioned and replaced with larger combined generic medical/cardiac precincts called hybrid units. Hybrid units are staffed with a low proportion of specialist critical care nurses. These changes may pose risks to nurse satisfaction and retention, and quality of patient care. The aims of this study were to explore specialist cardiac nurses’ perceived work satisfaction across four CCUs, and differences in satisfaction between dedicated and hybrid CCUs.

**Methods:**

This concurrent mixed methods study comprised two Phases in four Victorian CCUs (2 dedicated, 2 hybrid). In Phase 1, 74 specialist cardiac nurses completed the Professional Practice Environment (PPE) Scale. In Phase 2, 17 specialist cardiac nurses were interviewed to further explore elements of the PPE subscales. Descriptive, inferential (Phase 1), and content analyses (Phase 2) were performed.

**Results:**

Survey participants’ median age was 38 years (IQR 30, 45). The median PPE Scale score was 3.10 (IQR 2.90, 3.10) indicating high levels of satisfaction with their workplaces. Specialist cardiac nurses in one hybrid unit were significantly less satisfied compared with each of the other three units (*p* < 0.05). There were no significant differences in overall satisfaction or in any subscale of the PPE Scale between dedicated and hybrid units. Qualitative data revealed nurses in hybrid units felt they had less control over practice, lacked autonomy, had poor relationships with physicians, and experienced inadequate nurse leadership.

**Conclusions:**

Specialist cardiac nurses’ workplace satisfaction overall is high, with no significant differences between dedicated and hybrid CCUs. However, the structure of specialist cardiac units and NUM leadership skill level can impact nurses’ satisfaction with their workplace and collegial relationships. Strong nursing leadership that is respectful of nursing expertise and places patient safety foremost positively impacts nurses’ satisfaction. Further studies should assess the impact of the types of CCUs and NUM leadership on workforce factors such as nurse retention rates and patient outcomes such as adverse events.

## Background

Dedicated Coronary Care Units (CCUs) are defined as units with a high proportion (> 60%) of nurses with specialist qualifications that admit predominantly cardiac patients. Dedicated CCUs have been shown to reduce case-fatality rates associated with cardiovascular disease [1–3]. International guidelines endorse this model of care [4]. However, in Victoria, Australia, several dedicated CCUs have been decommissioned and replaced with larger combined generic medical/cardiac precincts [5]. There is much heterogeneity in Victorian CCU models; CCUs are commonly merged with cardiology wards, day procedure units, Intensive Care Units (ICU), and High Dependency Units [5, 6]. Accordingly, many CCU beds are occupied by patients whose primary diagnosis is a non-cardiac condition [5].

Hybrid CCUs tend to have a lower proportion of nurses with postgraduate critical care or cardiac/coronary care qualifications compared to dedicated CCU models. This is despite international guidelines recommending > 75% of the workforce in all CCUs comprise critical care qualified nurses [4, 7]. The change from dedicated to hybrid CCU models has been attributed to advances in the management of cardiac patients such as percutaneous coronary interventions and reduced lengths of stay [5]. Yet, there exists minimal evidence to suggest that processes, nursing care delivery or patient outcomes are actually enhanced by such restructuring endeavours [8].

Structural unit changes in cardiac care delivery have raised concerns about the quality and safety of patient care in Australian CCUs. The need to preserve a quality practice environment in order to optimise patient safety is clearly articulated in professional standards from the Nursing and Midwifery Board of Australia [9] and the National Safety and Quality Health Service Standards from the Australian Commission on Safety and Quality in Health Care [10]. High proportions of in-hospital adverse events have been associated with stressful practice environments, unreasonable workloads, and poor clinician expertise [11]. Demands on CCUs are likely to rise with increasing burden of cardiovascular disease and chronic comorbidities associated with an ageing population [4]. Thus, cardiac nursing expertise provided in dedicated CCUs will be in high demand; hybrid CCUs do not provide such nursing expertise for all admitted cardiac patients.

A meta-analysis by Kane et al. [12] found that increased presence of critical care qualified staff significantly reduces respiratory failure, cardiac arrest, and death. Another meta-analysis by Driscoll et al. [13] found that higher nurse staffing levels in acute specialist units such as critical care, intensive care, and coronary care, decreased the risk of in-hospital mortality by 14% (0.86, 95% CI 0.79–0.94). Further, in ICUs where there are fewer registered nurses with specialist critical care qualifications patients are at greater risk of poor outcomes [14, 15]. Inadequate nursing care for patients with acute coronary syndrome has been linked to poor patient outcomes and heightened mortality [16]. The Joint Commission on Accreditation of Healthcare Organizations [17] found that almost 25% of adverse events that occurred in acute care settings in the United States of America and resulted in profound injury or death were associated with inexperienced and/or inadequate levels of nursing staff.

The workforce of hybrid CCUs may comprise just 24–35% qualified specialist cardiac nurses [5]. The ramifications of this skill mix for both nurses and patient outcomes is inextricably linked. In hybrid CCUs, specialist cardiac nurses may risk deskilling and/or job dissatisfaction as there may be fewer opportunities to care for high acuity cardiac patients or they may have unreasonably high workloads due to the high proportion of general nurses; there may also be added pressure on ICUs to admit higher acuity patients [5]. Job dissatisfaction has the potential to result in attrition of specialist cardiac nurses in CCUs, in turn, reducing inexperienced nurses’ access to valuable clinical mentorship, the appeal of cardiac nursing, and the quality of patient care [6].

Maintaining a satisfied nursing workforce is crucial to patient safety. Currently, the specific impact of the type of CCU model (dedicated or hybrid) on nursing job satisfaction is poorly understood. The aims of this study were to explore specialist cardiac nurses’ perceived work satisfaction across four CCUs, and differences in satisfaction between dedicated and hybrid CCUs.

## Methods

### Design

Ethics approval for the study was granted from Deakin University (2014–049) and from the Human Research in Ethics Committee at each of the four hospitals (559/12, 13/108, LR40/1213, 2012.211). A concurrent mixed methods design with two Phases was used. In Phase 1, demographic information and specialist cardiac nurses’ perceptions of the clinical environment in CCUs was collected with a survey. After Phase 1, a subset of nurses were interviewed in Phase 2 using semi-structured individual or focus-group interviews to explore their perspectives on their workplace.

### Setting

Four Victorian CCUs, including two dedicated (Units A and B) and two hybrid (Units C and D) CCUs from three metropolitan hospitals (Unit A, C and D) and one regional hospital (Unit B), were involved in this research. All were level 2 units with infrastructure to treat high risk, high acuity cardiac patients [18]. Dedicated CCUs have a high proportion (> 60%) of nurses with specialist qualifications and admit predominantly cardiac patients. Hybrid CCUs have lower proportions of nurses with specialist qualifications and accept a mixture of patients, even those with non-cardiac diagnoses.

### Participants

In Phase 1, all registered nurses working within the CCUs at the four hospitals were invited to participate in the survey. Nurses were eligible if they

- worked more than 8 h per week,
- held a postgraduate critical care or coronary care qualification, and
- were permanently employed in the CCU.

Specialist cardiac nurses were invited to participate via flyers for both Phases, and advocacy by managers of the unit. Participant specialist cardiac nurses completed the paper-based surveys that were placed in CCUs. Responses were returned via post or a box left for that purpose in each CCU; return of the survey implied informed consent. A separate page of the survey invited specialist cardiac nurses to indicate their willingness or not to be approached to participate in Phase 2. Volunteers provided their contact details to researchers who set up either a focus group or individual interview as per the participant’s choice on the survey form. Written informed consent was obtained prior to the commencement of the interviews. All specialist cardiac nurses were assured that their data would be deidentified.

### Data collection instruments

The Phase 1 survey collected data on demographics, work experience and qualifications, and responses to the Professional Practice Environment (PPE) Scale. The PPE scale evaluates nursing perceptions of the work environment via 38 items in eight subscales with responses on a Likert-scale from 1 (strongly disagree) to 4 (strongly agree) [19, 20]. The eight subscales comprise: handling disagreement and conflict (items 21–28); internal work motivation (items 29–35); control over practice (items 5–14); leadership and autonomy in clinical practice (items 1–3, 9, and 12); staff relationships with physicians (items 4 and 13); teamwork (items 17–20); cultural sensitivity (items 36–38); and communication about patients (items 15 and 16). Versions of the PPE Scale have been validated in the American and Australian acute care settings, and in the Australian general practice setting [19–22]. Though we did not identify records of its use in the Australian CCU setting specifically, coronary care nursing experts (JC and AD) confirmed the relevance of the PPE Scale to the Australian coronary care context. The PPE Scale originated from studies conducted in the United States surrounding Magnet Hospitals [19]. Magnet Hospitals are those which are said to have an appealing working environment and as such, are associated with high levels of staff satisfaction and retention [19].

Semi-structured interviews were conducted by a Research Assistant (AC) in a private location within each hospital or by phone. During the interviews, specialist cardiac nurses were first asked open-ended questions about their level of workplace satisfaction generally. The subscale domains in the survey were then used as prompts to explore issues affecting specialist cardiac nurses’ workplace satisfaction; e.g. whether they were satisfied with their relationships with physicians, and their perceived control over nursing practice. Comments regarding factors positively or negatively impacting their work satisfaction were invited. Interviews were 20–40 min in duration. Data saturation was reached when no further new information was provided by participants regarding workplace satisfaction issues. Analysis of Phase 1 data did not inform the interview questions in Phase 2.

### Data analysis

Data from Phase 1 were analysed using SAS (version 9.4, SAS Institute). Demographic data were summarised using median and interquartile range or frequency and percentage.

Each item in the PPE Scale was scored in accordance with recommendations; selected items were reversed [19–22]. Each subscale score was calculated as the median of the corresponding items. A higher subscale score indicated a greater level of satisfaction. We report medians and interquartile ranges for each subscale of the PPE and for the total score.

The PPE subscale scores showed a non-symmetric distribution for some of the centres, so scores between hybrid and dedicated CCU types and across centres were compared using the Kruskal-Wallis test. The same test was used for pairwise comparisons; Bonferroni adjusted *p*-values are reported. We further fitted a general linear model for the only variable with an approximately normal distribution, PPE overall score, adjusting for specialist cardiac nurses’ characteristics that showed a difference between units (working permanent rostered days; nurses’ age; years qualified as a registered nurse; years qualified as a critical care/coronary care nurse). The proportion of shifts allocated to high acuity cardiac patients was not included in the model because this variable is highly related to the type of unit.

The interviews from Phase 2 were transcribed and thematically analysed using a content analysis approach documented by Elo and Kyngӓs [23]. Themes were informed by the eight sub-scales of the PPE Scale. Data credibility was addressed by one research assistant (AC) interviewing participants and transcribing the interviews verbatim. Data familiarisation processes involved reading and rereading transcripts, initially coding data, and deriving categories and emergent themes (AC and AD). Codes and themes were discussed, critiqued and further refined by two members of the research team (SS and JC). Where relevant, specific comments in relation to dedicated and hybrid CCU models were noted. These processes provided overall agreement that the findings were representative of the data and that trustworthiness was established.

## Results

### Participant characteristics

A total of 74 specialist cardiac nurses returned the survey (Unit A = 23; Unit B = 19; Unit C = 16; Unit D = 16); 59 (79.7%) of those completed the PPE Scale in its entirety. Participant demographics are presented in Table 1. Overall, the median age was 38 years (IQR 30, 45). Participants had a median of 8 years (IQR 4, 16) experience as qualified specialist critical care/coronary care nurses. The median length of time employed in their current unit was 5 years (IQR 2, 10). Participant work patterns are also presented in Table 1. There were significant differences between units for specialist cardiac nurses rostered permanent days (*p* = 0.006); specialist cardiac nurses receiving less than 25% of their shifts allocated to high acuity cardiac patients (p = < 0.001); years qualified as registered nurses (*p* = 0.001) and years qualified as critical care/coronary care nurses (*p* = 0.002). Specific significant pairwise comparisons between units are also noted in Table 1.

**Table 1:** *Participant Demographic Characteristics and Work Patterns*

	Overall (*N* = 74)	Unit A (*n* = 23)	Unit B (*n* = 19)	Unit C (*n* = 16)	Unit D (*n* = 16)	*p*-value
	*n (%)*	*n (%)*	*n (%)*	*n (%)*	*n (%)*	
Registered Nurse	32 (43%)	6 (26%)	6 (32%)	9 (56%)	11 (69%)	0.148
Clinical Nurse Specialist	20 (27%)	9 (39%)	7 (37%)	2 (13%)	2 (13%)	
Associated Nurse Unit Manager	19 (26%)	7 (30%)	5 (26%)	5 (31%)	2 (13%)	
Clinical Nurse Educator	3 (4%)	1 (4%)	1 (5%)	0 (0%)	1 (6%)	
Day time shifts only	6 (8%)	2 (9%)	2 (11%)	2 (13%)	0 (0%)	0.426
Night shifts only	3 (4%)	0 (0%)	2 (11%)	1 (6%)	0 (0%)	
Rotating roster (days and nights)	61 (82%)	20 (87%)	15 (79%)	11 (69%)	15 (94%)	
Permanent shifts unspecified type	4 (5%)	1 (4%)	0 (0%)	2 (13%)	1 (6%)	
Permanent days worked (a)	14 (19%)	1 (4%)	5 (26%)	7 (44%)	1 (6%)	0.006
Proportion of shifts allocated to high acuity cardiac patients						
< 25% (b)	17 (23%)	1 (4%)	7 (37%)	3 (19%)	6 (38%)	< 0.001
25–50%	13 (18%)	5 (22%)	1 (5%)	5 (31%)	2 (13%)	
50–75%	23 (31%)	9 (39%)	10 (53%)	4 (25%)	0 (0%)	
100%	21 (28%)	8 (35%)	1 (5%)	4 (25%)	8 (50%)	
	Median (IQR)	Median (IQR)	Median (IQR)	Median (IQR)	Median (IQR)	
Age (years) (c)	38 (30, 45)	40 (32, 45)	44 (37, 52)	34 (29, 41)	34^a^ (26, 38)	0.012
Years qualified as RN (d)	12 (5, 20)	13 (6, 22)	21 (10, 30)	7 (4, 14)	7 (2, 15)	0.001
Years qualified as CCRN (e)	8 (4, 16)	12 (4, 20)	12.5^d^ (8, 28)	3^b^ (2, 5)	5.5^c^ (3, 9)	0.002
Hours worked per week	32 (28, 38)	32 (20, 36)	32 (20, 40)	36 (31, 39)	32 (32, 40)	0.116

*Note: IQR* Interquartile range, *RN* Registered nurse, *CCRN* Critical care registered nurse; ^a^ 3 missing data; ^b^ 4 missing data; ^c^ 8 missing data; ^d^1 missing data. Significant pairwise comparisons between units (adjusted *p*-values): (a) Unit A vs Unit C (*p* = 0.027); (b)

Unit A vs Unit D (*p* = 0.015), Unit A vs Unit B (*p* = 0.032), Unit B vs Unit D (*p* = 0.002); (c) Unit B vs Unit D (p = 0.032); (d) Unit B vs Unit C (*p* = 0.024), Unit B vs Unit D (*p* = 0.008); (e) Unit A vs Unit C (*p* = 0.030), Unit B vs Unit C (*p* = 0011)

### The PPE scale

A summary of the scores for each subscale of the PPE Scale, overall and by unit, is displayed in Table 2. Median values for all eight subscales were above 2.90. The overall median was 3.10 (IQR 2.90, 3.10) indicating specialist cardiac nurses were satisfied with their workplace. Nurses’ PPE satisfaction scores did not differ significantly according to whether they worked in a hybrid or dedicated CCU. Specialist cardiac nurses in one hybrid unit, Unit C, reported significantly lower overall PPE satisfaction scores compared with each of the other three units (all comparisons, *p* < 0.05). Specialist cardiac nurses in Unit C were significantly less satisfied than colleagues in one dedicated unit (A) and the other hybrid unit (D) regarding ‘Relationships with Physicians’ (all comparisons, *p* < 0.05); and less satisfied than colleagues in Units A and B for the PPE subscales of ‘Control Over Practice’ and ‘Leadership’ (both *p* < 0.05) (see Table 2). When we adjusted for specialist cardiac nurses’ characteristics that were different across units (working permanent rostered days; nurses’ age; years qualified as a registered nurse; years qualified as a critical care/coronary care nurse), nurses’ overall satisfaction was significantly lower in Unit C than all other units (p < 0.05 all comparisons).

**Table 2:** *Professional Practice Environment Scale Scores*

	Overall (N = 74)	Unit A (*n* = 23)	Unit B (*n* = 19)	Unit C (*n* = 16)	Unit D (*n* = 16)	*p*-value
	Median (IQR)	Median (IQR)	Median (IQR)	Median (IQR)	Median (IQR)	
Overall median^a^	3.1 (2.9, 3.1)	3.2 (3.0, 3.2)	3.1 (3.0, 3.2)	2.7 (2.6, 2.8)	3.2 (3.0, 3.1)	0.011
1. Handling disagreement and conflict	2.9 (2.7, 2.9)	3.0 (2.8, 3.0)	2.9 (2.9, 3.1)	2.6 (2.3, 2.6)	3.0 (2.9, 3.0)	0.054
2. Internal work motivation	3.3 (3.0, 3.3)	3.3 (3.0, 3.4)	3.3 (3.0, 3.3)	2.9 (2.6, 3.0)	3.4 (3.0, 3.4)	0.125
3. Control over practice^b^	3.0 (2.7, 3.0)	3.1 (2.9, 3.1)	3.1 (3.0, 3.2)	2.6 (2.4, 2.6)	3.0 (2.7, 3.0)	0.001
4. Leadership and autonomy in clinical practice^c^	3.2 (3.0, 3.2)	3.4 (3.0, 3.4)	3.2 (3.0, 3.2)	2.9 (2.8, 2.9)	3.4 (2.9, 3.2)	0.004
5. Staff relationships with physicians^d^	3.0 (3.0, 3.2)	3.0 (3.0, 3.2)	3.0 (3.0, 3.1)	3.0 (2.5, 2.9)	3.5 (3.0, 3.5)	0.006
6. Teamwork	3.0 (2.8, 2.9)	3.0 (2.8, 3.0)	3.0 (2.8, 3.0)	2.8 (2.8, 2.8)	3.0 (2.8, 2.9)	0.465
7. Cultural sensitivity	3.0 (3.0, 3.2)	3.0 (3.0, 3.1)	3.0 (3.0, 3.3)	3.0 (2.8, 3.0)	3.0 (3.0, 3.3)	0.227
8. Communication about patients	3.0 (3.0, 3.2)	3.0 (3.0, 3.2)	3.0 (3.0, 3.3)	3.0 (3.0, 3.1)	3.0 (3.0, 3.3)	0.402

*Note: IQR* Interquartile range. Significant pairwise comparisons between units (adjusted *p*-values): ^a^Unit C vs Unit A (*p* = 0.022), Unit C vs Unit B (*p* = 0.029), Unit C vs Unit D (*p* = 0.044); ^b^Unit C vs Unit A (*p* = 0.004,) Unit C vs Unit B (*p* = 0.006); ^c^Unit C vs Unit A (*p* = 0.004), Unit C vs Unit B (*p* = 0.035); ^d^ Unit C vs Unit A (*p* = 0.006), Unit C vs Unit D (*p* = 0.029), Unit B vs Unit D (*p* = 0.029)

### Interviews

Of the 74 specialist cardiac nurses who participated in Phase 1, 28 from all CCUs initially offered to be interviewed in Phase 2. Seventeen specialist cardiac nurses were interviewed (Dedicated *n* = 11; Hybrid *n* = 6), the rest were not available due to rostering schedules during the time when the interviews were planned, i.e. the month post survey. There were 13 individual interviews and one focus group consisting of four specialist cardiac nurses. Three individual participants were interviewed by phone at their preference; the remaining interviews were face-to-face and conducted in private workplace rooms.

#### Handling disagreement and conflict

Specialist cardiac nurses perceived the handling of disagreements and conflicts to be very important to their job satisfaction. Where disagreements were not addressed or resolved in a timely manner, negative feelings and a sense of carrying a burden resulted. Disagreements were reported between specialist cardiac nurses and nurse unit managers (NUMs); specialist cardiac nurses and medical staff; and between colleagues. Most disagreements arose within the nurse-NUM relationship, resulting in specialist cardiac nurses’ daily work life becoming very stressful. Specialist cardiac nurses reported poor communication and teamwork skills by the NUM, and in hybrid units the NUM was viewed as not approachable, a poor listener or not a representative of specialist cardiac nurses’ views. These concerns were most pronounced among experienced specialist cardiac nurses who felt their knowledge and skills were undermined by the NUM, especially in Unit C. One nurse explained:

“ *… if you are someone who is good at your job, or who is willing to challenge and put forth opinions that may try and improve the unit, she will view you as a threat and she will make your life very, very difficult”.*

In the nursing-medical relationship, issues most commonly arose when specialist cardiac nurses could not contact registrars and residents easily to discuss concerns about patients. Specialist cardiac nurses recognised doctors were inundated with work but nonetheless expected timely responses to paged messages about patient care.

A few specialist cardiac nurses reported friction between colleagues. Specialist cardiac nurses recognised that they had to work with each other and share patient responsibilities especially when colleagues had to leave the unit to transport patients or take meal breaks. In one hybrid unit, associate NUMs considered it important to understand nurses’ personalities, experience, and rivalries when preparing rosters. Although unpleasant, specialist cardiac nurses did not believe conflicts impacted on care delivery.

There were no clear processes for addressing and resolving disputes in any unit. Nurses tended to solve their own problems, sometimes bypassing the NUM when there were poor nurse-NUM relationships which was more frequent in hybrid units. Inexperienced nurses typically consulted more experienced specialist cardiac nurses for advice, whereas experienced specialist cardiac nurses consulted senior doctors. Specialist cardiac nurses typically consulted their NUM only if there was an issue with medical and/or nursing care. Conflicts generally remained unresolved and specialist cardiac nurses often chose to be professional and solve it themselves, live with it or ignore it.

*“I think that the expectation of not only myself but other staff will be to resolve professional problems internally and individually.”*

#### Internal work motivation

Specialist cardiac nurses acknowledged their careers suited people passionate about helping others. Specialist cardiac nurses reported feelings of satisfaction and gratification when their efforts enhanced patient care and improved outcomes. All nurses expressed commitment to their role, placing patients at the centre of their practice. This notion helped to put aside stress related to personal differences or poor resource availability for instance, and maintain focus on the delivery of quality patient care. This motivation was internal and self-generated. However, specialist cardiac nurses mentioned a positive, encouraging, progressive and overall happy unit did increase their job satisfaction.

#### Control over practice

Specialist cardiac nurses in all units voiced concerns about the overall image of cardiac nursing, particularly in relation to nurses in ICUs or emergency departments. Specialist cardiac nurses in dedicated units felt dissatisfied about their level of influence on care practices beyond the CCU walls, and frustrated by a lack of knowledge by those outside CCU about their knowledge and skill, their speciality, and the high acuity of patients. Specialist cardiac nurses in hybrid CCUs thought they were looked upon as step down units, temporary short stay units or high dependency units. Consequently, there was no strong or unique identity within the hospital. Insufficient status in the organisation was mentioned by specialist cardiac nurses in all CCUs:

*“I don’t think they (other ward staff) have any idea of what we actually do or, you know, have an understanding of the type of patients we have and get and the acuity that we have. They probably don’t respect the role that we have. I don’t think they have much knowledge about coronary care”. (Nurse from dedicated unit)*

*“ … there is a lot of pressure … patients get moved quite frequently to make room for others. If we get a call from the emergency department, say there is a STEMI, you have to make room. You have to find the space for them. So someone who will still be considered a coronary care patient, they have to move to make room.” (Nurse from hybrid unit)*

The hybrid CCU model was perceived to be a more stressful environment at times and this took away some satisfaction associated with delivering patient care. Experienced specialist cardiac nurses working in hybrid units felt their coronary care and high acuity skills were underutilised when frequently caring for non-cardiac patients who did not need their expertise. Specialist cardiac nurses in hybrid units had to work under considerable pressure dictated by high patient flow rates which led to dissatisfaction. This was compounded by specialist cardiac nurses being unable to provide high acuity cardiac patients with evidence-based comprehensive education and such patients being allocated to very experienced staff for care delivery. Experienced specialist cardiac nurses expressed sadness and frustration with a perceived lower quality of care delivery compared to their earlier years in dedicated units. A patient access nurse position, with the sole role of managing patient admissions and discharges in and out of the unit, existed in one hybrid unit to buffer the impact of rapid bed turnaround and patient flow. This position was viewed positively because there were many inexperienced nurses who could not speak with authority to influence others to balance patient flow with the provision of appropriately skilled nurses for patients. A comment from a senior specialist cardiac nurse reflects this view:

*“When there is a large number of critical care students working in the acute section, I don’t feel that they have the control over practice … when I am doing that patient access role [a nurse who coordinates the unit admission and discharge of patients], one of my first thoughts is how do I keep this safe? How do I keep it reasonable for nurses at the bedside to deliver the care?” (Nurse in hybrid unit)*

Specialist cardiac nurses in dedicated units thought their structure encouraged slightly more control over practice which they felt would result in better outcomes for the patients. One specialist cardiac nurse commented that:

*“having speciality nurses for a speciality care and having a speciality patient subgroup that they are trained to care for, the ward is setup for, can only result in better outcomes* ***.”***

Some experienced specialist cardiac nurses thought resources had dwindled due to cost cutting over which they had no control. Concerns about wasting nursing time and compromising patient care due to poor quality consumables and equipment, such as insufficient or broken intravenous infusion pumps were raised.

#### Leadership and autonomy in clinical practice

Specialist cardiac nurses from all but hybrid Unit C were very satisfied with their level of leadership and autonomy because they utilised their clinical knowledge and skills during patient care. Specialist cardiac nurses recognised the importance of being afforded the necessary freedom to exercise their judgement in a timely fashion. Specialist cardiac nurses enjoyed delivering patient care, whether that be assessing or monitoring patients; analysing or interpreting data from ECGs or closely observing patients for changes in their condition. Specialist cardiac nurses consulted doctors directly with concerns and could initiate prompt treatment when appropriate.

*“Working in CCU, I think I have a reasonable amount of autonomy to assess our patients, to make calls for our patients, to be involved in the treatment process, and identifying and changing things as issues come up.” (Nurse from dedicated unit)*

In one dedicated unit, protocols enabled specialist cardiac nurses to intervene more autonomously at the bedside; they could adjust various treatments via protocols pre-signed by doctors on admission. This responsibility heightened their vigilance, sense of professionalism, and satisfaction. Autonomy was considered vital for themselves and patient care:

*“If we don’t have any autonomy in terms of power and ability to practise, to engage to think, to contribute to outcomes, then I think it would decrease our involvement, it would stifle initiatives, it will decrease our clinical skills … we’d become task-orientated.” (Nurse from dedicated unit)*

Specialist cardiac nurses in one hybrid unit expressed concern regarding limited opportunities to progress in leadership roles. The NUM was considered an important determinant in providing opportunities and encouragement for specialist cardiac nurses to develop leadership skills.

“*Leadership development isn’t well thought out … there is a role called patient access nurse that involves bed flow during the day … but when that nurse is on sick or annual leave, the opportunity for others to do that role isn’t really given. The NUM usually steps in. So our development isn’t particularly seen as important*.” *(Nurse from hybrid unit)*

#### Staff relationships with physicians

All specialist cardiac nurses believed that relationships and communication with doctors was very important; this influenced the morale of specialist cardiac nurses and the quality of patient care. They felt valued and respected when doctors of all designations sought their opinion and listened to them. Common friction occurred between specialist cardiac nurses and residents due to residents’ lack of respect for specialist cardiac nurses’ experience. Participants reported doctors could be quite brash in their approach to nurses.

Senior specialist cardiac nurses felt that the longer clinicians were associated with the hospital the more enduring their relationships were; this was reflected in nurses’ positive relationships with consultants. Specialist cardiac nurses who had worked in the unit for a long time had strong relationships with other clinicians, and could communicate their concerns in an open manner. Senior specialist cardiac nurses acknowledged junior nurses may not have the confidence or knowledge to express their concerns so readily.

Specialist cardiac nurses from dedicated CCUs expressed better relationships and communication paths between doctors and nurses. This arose because the structure facilitated formation of long-term relationships and more open lines of communication as both disciplines worked exclusively in the area. In the hybrid CCUs, the variety of patient conditions meant that there were several teams of doctors to liaise with, making it difficult to form relationships with multiple large medical teams.

#### Teamwork

The NUM was perceived to play a critical role in encouraging teamwork. When the NUM created and worked within a team spirit, mutual trust and respect existed. Both NUMs of hybrid units were not perceived to create this atmosphere which was a source of stress and frustration because nurses wanted teamwork to be encouraged. Experienced specialist cardiac nurses in these hybrid units believed that the model of care was not conducive to teamwork. During the restructure to a hybrid model, many senior specialist cardiac nurses resigned with junior nurses replacing them. Senior specialist cardiac nurses felt that these nurses had little awareness of the happenings in the rest of the unit and were not competent or mature enough to help others.

Aside from the NUM comments, specialist cardiac nurses thought they practiced good teamwork. While specialist cardiac nurses were allocated specific patients during a shift, most nurses recognised that they also worked in a team. In one dedicated CCU, specialist cardiac nurses attended hospital Medical Emergency Team (MET) calls; hence their colleagues provided care to their patients during their absence which ranged from minutes to hours. The high patient acuity and high patient turnover due to numerous patients admitted post procedures in hybrid units meant situations could change very quickly and cause stress. Unless nurses trusted and supported each other to put patient interests first, it was not possible to deliver quality patient care.

#### Communication about patients

All specialist cardiac nurses thought communication about patients was essential to their role in delivering quality patient care. There was some disappointment among specialist cardiac nurses from hybrid units that specialist cardiac nurses were no longer necessarily present on the ward rounds. They found this move detrimental to communication about patients and their own learning.

*“I think the unit is definitely not as good as it used to be. For instance, the nurses used to present patients on ward rounds in the unit so as a nurse we used to relay information about the patient to the doctor, they were asking you questions about their care. Doesn’t seem to happen any more if at all.” (Nurse from hybrid unit)*

Even though most specialist cardiac nurses viewed bedside handover processes positively, some had hesitations. Earlier handover practices involved all specialist cardiac nurses knowing all patients and specialist cardiac nurses found this useful when they needed to cover breaks and deliver care to colleagues’ patients.

“*So they are now focusing on a bedside handover … which is speedier, gives you an opportunity to go in and see the patients and check things out in depth maybe a bit more. But I have no idea whatsoever who the other nurses are. Whereas before … I would know all of them quite well. What if someone goes off to the MET call?” (Nurse from dedicated unit)*

One hybrid unit employed clinical support nurses each shift whose primary role was to address nurses’ concerns about patient care before calling doctors. Experienced specialist cardiac nurses viewed this positively as it streamlined the process for managing concerns but acknowledged junior nurses may feel undervalued as they were not granted sufficient authority to contact doctors directly.

## Discussion

In this mixed method study, types of CCUs and their impact on the specialist cardiac nursing workforce were explored specifically in relation to job satisfaction. The PPE Scale was used to assess specialist cardiac nurses’ perceptions of their current practice environment (Phase 1) and interviews were conducted to explore issues related to the PPE subscales as well as other areas of satisfaction or concern (Phase 2).

The major finding of this study was that the results of the PPE Scale indicated that overall, specialist cardiac nurses in all CCUs were satisfied with their work environments. Second, specialist cardiac nurses in Unit C, a hybrid CCU, were significantly less satisfied than those in the other three units; however, no statistically significant differences in satisfaction were found between hybrid and dedicated units. The difference between Unit C and the dedicated units remained after adjusting for specialist cardiac nurses’ characteristics.

This study also found that specialist cardiac nurses in Unit C were significantly less satisfied than others on the specific PPE subscales of ‘Control Over Practice’, ‘Leadership and Autonomy in Practice’, and ‘Staff Relationships with Physicians’. The potential for job dissatisfaction secondary to significant restructuring may negatively influence recruitment, retention [24, 25], and patient mortality [26]. A high turnover of staff is costly and may negatively influence morale, team dynamics, and therefore quality of care [24, 27, 28]. These are critical considerations for the longevity of the nursing workforce and patient safety. Given further nursing shortages are forecast, particularly in acute and critical care areas [29], ensuring nurses in all cardiac units feel satisfied with their work is essential to the profession and the community.

The content analysis of the interview data highlighted that specialist cardiac nurses in dedicated units expressed greater satisfaction regarding control over their practice, and experienced leadership and autonomy in their work because they used their clinical expertise regularly. However, all respondents thought specialist cardiac nurses had insufficient intra-organisational status and professional identity as specialist cardiac nurses beyond the CCU walls. For those in hybrid units, this feeling was compounded by caring for patients who did not have specialist cardiac needs, and repeatedly having to care for new patients in a high flow environment. Unlike cardiac nurses, Australian critical care and emergency nurses have peak professional advocacy bodies, both of which publish standards for practice to clearly set expectations of their workforce for patient care delivery [30, 31].

When experienced specialist cardiac nurses in hybrid units were not allocated to high acuity patients requiring their expertise, they were concerned about deskilling, and felt work dissatisfaction plus a lower professional self-esteem. Senior specialist cardiac nurses were also concerned for patient care in relation to the widening gap in skill mix due to less experienced or qualified cardiac nurses working in hybrid units. As shown by Aitken et al. [32], having sufficient and appropriately qualified nurses for patients impacts patient safety. Further, there are few postgraduate courses specifically educating nurses for current cardiac workforce needs [33]. However, given the ageing population and high incidence of cardiac disease [34], there will be a commensurate need for more specialist cardiac nurses in the near future.

This study also found that specialist cardiac nurses highly valued the physician-nurse relationship, teamwork with colleagues, and communication channels for the provision of high quality care. However, specialist cardiac nurses in hybrid units were less satisfied with these relationships due to the multiple medical teams and short rotations of junior doctors. Further, specialist cardiac nurses in hybrid units expressed dissatisfaction with their NUMs due to poor leadership behaviours regarding teamwork and communication channels, and failing to offer staff leadership opportunities or represent cardiac nursing as highly specialised. This dissatisfaction in respect to leadership behaviours was notably not captured in the results of the PPE Scale; reasons for this are unclear. However, it may be that when the specialist cardiac nurses were afforded time to expand on their thoughts and feelings in the interviews in their own words, there were other relevant points to explore which were not perceived to match the items about leadership, teamwork, and communication in the PPE Scale. The interview questions may have also assisted participating nurses to better understand the areas for discussion. Teamwork and communication within and between disciplines are vital for patient safety. Early work by Estabrooks et al. [26] in cardiac patients demonstrated that mortality rates decreased when nurses had a higher education level (OR = 0.81, 95%CI = 0.68–0.96); an increased skill mix existed (OR = 0.83, 95%CI =0.73–0.96), and there were strong nurse-physician relationships (OR = 0.74, 95%CI =0.60–0.91). Thus, concerted efforts are required by nurses and their leaders to more effectively manage team dynamics and communication. Nurses’ career intentions regarding staying in CCU were not explored in this study. The inclusion of such or follow up is warranted.

### Study limitations

A strength of this study was the use of a validated tool combined with interviews which allowed participants to inform the study freely or expand upon key issues raised in their PPE survey. Interview findings supported the quantitative findings that specialist cardiac nurses in the hybrid units, particularly Unit C, were less satisfied with their workplace. Although four sites were chosen, the sampling method and size may have contributed to results. The study was done at a single point in time, thus longitudinal studies may provide more insights into whether and how specialist cardiac nurses’ satisfaction with their workplace changes over time.

## Conclusions

In this concurrent mixed methods study, experienced specialist cardiac nurses in dedicated and hybrid CCUs were found to be satisfied in their workplace; however, specialist cardiac nurses in one hybrid unit were significantly less satisfied in the domains of Control over Practice, Leadership and Autonomy in Clinical Practice, and Staff Relationships with Physicians. Specialist cardiac nurses expressed concerns about the lack of professional recognition provided to specialist cardiac nurses which was compounded by hybrid unit NUMs who were perceived to provide poor leadership, communication, and teamwork skills within and beyond the CCU walls. This study offers new insights into some of the negative impacts of restructuring specialist cardiac units on the satisfaction of the specialist coronary care nursing workforce. Nursing job dissatisfaction in hybrid CCUs must be addressed as an exodus of experienced specialist cardiac nurses will have profound consequences for the future of specialty nursing practice and the quality of patient care. Future research should analyse adverse event data to investigate potential relationships between skill mix, PPE Scale scores, and type of CCU.

## Data Availability

The datasets generated and/or analysed during the current study are not publicly available as ethical restrictions precluded the publication of raw data.

## Abbreviations

CCU: Coronary Care Unit
ICU: Intensive Care Unit
MET: Medical Emergency Team
NUM: Nurse Unit Manager
PPE: Professional Practice Environment
